# Medications influencing the risk of fall-related injuries in older adults: case–control and case-crossover design studies

**DOI:** 10.1186/s12877-023-04138-z

**Published:** 2023-07-22

**Authors:** Yu-Seon Jung, David Suh, Eunyoung Kim, Hee-Deok Park, Dong-Churl Suh, Sun-Young Jung

**Affiliations:** 1 Chung-Ang University College of Pharmacy, 84 Heukseok-Ro, Dongjak-Gu, Seoul, South Korea; 2 School of Public Health, University of Michigan, Ann Arbor, MI USA; 3 Rutgers, The State University of New Jersey School of Pharmacy, 160 Frelinghuysen Rd, Piscataway, NJ USA

**Keywords:** Fall-related injuries, Fall risk-increasing drugs, Claims database, Case–control, Case-crossover

## Abstract

**Background:**

Medications influencing the risk of fall-related injuries (FRIs) in older adults have been inconsistent in previous guidelines. This study employed case–control design to assess the association between FRIs and medications, and an additional case-crossover design was conducted to examine the consistency of the associations and the transient effects of the medications on FRIs.

**Methods:**

This study was conducted using a national claims database (2002–2015) in Korea. Older adults (≥ 65 years) who had their first FRI between 2007 and 2015 were matched with non-cases in 1:2 ratio. Drug exposure was examined for 60 days prior to the date of the first FRI (index date) in the case–control design. The hazard period (1–60 days) and two control periods (121–180 and 181–240 days prior to the index date) were investigated in the case-crossover design. The risk of FRIs with 32 medications was examined using conditional logistic regression after adjusting for other medications that were significant in the univariate analysis. In the case-crossover study, the same conditional model was applied.

**Results:**

In the case–control design, the five medications associated with the highest risk of FRIs were muscle relaxants (adjusted odd ratio(AOR) = 1.35, 95% confidence interval (CI) = 1.31–1.39), anti-Parkinson agents (AOR = 1.30, 95%CI = 1.19–1.40), opioids (AOR = 1.23, 95%CI = 1.19–1.27), antiepileptics (AOR = 1.19, 95%CI = 1.12–1.26), and antipsychotics (AOR = 1.16, 95%CI = 1.06–1.27). In the case-crossover design, the five medications associated with the highest risk of FRIs were angiotensin II antagonists (AOR = 1.87, 95%CI = 1.77–1.97), antipsychotics (AOR = 1.63, 95%CI = 1.42–1.83), anti-Parkinson agents (AOR = 1.58, 95%CI = 1.32–1.85), muscle relaxants (AOR = 1.42, 95%CI = 1.35–1.48), and opioids (AOR = 1.35, 95%CI = 1.30–1.39).

**Conclusions:**

Anti-Parkinson agents, opioids, antiepileptics, antipsychotics, antidepressants, hypnotics and sedatives, anxiolytics, muscle relaxants, and NSAIDs/antirheumatic agents increased the risk of FRIs in both designs among older adults. Medications with a significant risk only in the case-crossover analysis, such as antithrombotic agents, calcium channel blockers, angiotensin II antagonists, lipid modifying agents, and benign prostatic hypertrophy agents, may have transient effects on FRIs at the time of initiation. Corticosteroids, which were only associated with risk of FRIs in the case–control analysis, had more of cumulative than transient effects on FRIs.

**Supplementary Information:**

The online version contains supplementary material available at 10.1186/s12877-023-04138-z.

## Background

The appropriate use of medication in older adults has become more crucial in geriatric care with the increasing aging population and polypharmacy. A fall occurs annually in 28–35% of adults older than 65 years worldwide, and medical costs associated with a fall in 2015 were approximately 50 billion dollars in the United States alone [1–3]. Fall-related injuries (FRIs) often require costly medical intervention in the short term. In the long term, older adults and their caregivers suffer significantly from a decreased quality of life from limited mobility, self-care ability, and overall health as well as anxiety/depression, which lead to early admission to long-term care facilities [4–7]. To prevent FRIs and maintain healthy lives among older adults, the appropriate use of medications and avoidance of prescribing medications that increase the risk of FRIs are necessary.

In clinical practice, however, it is often challenging to identify medications that increase the risk of FRIs because there is considerable variation in the consensus about which medications have FRI risks in published guidelines. All published guidelines recommend avoiding psychotropic medications and the broader category of central nervous system (CNS)-active medications in older adults [8–11]. In addition, vasodilators were included in the list of fall-risk drugs in the Screening Tool of Older Persons' Prescriptions and Screening Tool to Alert to Right Treatment (STOPP/START) criteria, and the Screening Tool of Older Persons Prescriptions in older adults with high fall risk (STOPPFall) criteria published in 2021 further agreed to include anticholinergics, diuretics, alpha-blockers (used as antihypertensives and for prostatic hyperplasia), centrally-acting antihypertensives, antihistamines, vasodilators (used in cardiac disease), and overactive bladder and urge incontinence medications as fall-risk drugs [9, 11].

Current guidelines are based on observational studies that have reported a consistent association of falls or FRIs with psychotropic medications but not with other medication classes [12–17]. The underlying causes for FRIs are multifactorial and include intrinsic factors as female, advanced age, and chronic conditions (i.e., arthritis, stroke, incontinence, and Parkinson’s disease), and extrinsic factors as environmental factors and medications [18–20]. The residual confounding for FRIs in the traditional case–control or cohort design and transient effects of medications at the initiation may have contributed to the inconsistent results in previous literature [21–23]. To address this inconsistency, employing both case–control and case-crossover designs, each with its own advantages and limitations, could be beneficial.

A case-crossover study is a self-controlled study design in which each patient serves as their own control, and this design is suitable for measuring short-term effects of transient exposure for immediate outcomes [21, 24, 25]. This design has the advantage of controlling for between-subject confounders, which is a common concern in the case–control design. On the other hand, a case–control study has the advantage of capturing both transient and cumulative effects of a drug, and it can compensate for the persistent bias in case-crossover analyses of chronic medications [21, 26]. Therefore, to resolve inconsistencies in previous studies on the falls or FRIS associated with medications, we conducted a population-based study to examine the association between medications and FRIs, as well as the transient effects of medications, by applying both study designs.

This study is the first population-based study conducted using case–control and case-crossover designs to investigate the associations of 32 medications with FRIs among older adults. The aim of this study is to examine the risk of FRIs associated with these medications using two study designs to assess consistency and transient effects. A traditional case–control design was conducted to identify medications that increase the risk of FRIs; in addition, a case-crossover analysis was employed to examine the consistency of the medications increased risks of FRIs in the case–control analysis by adjusting between-subject confounders and to investigate the transient effects of medications on FRIs.

## Methods

### Data source

This study used the National Health Insurance Service (NHIS)-senior cohort dataset (version 2.0), which consists of 511,953 individuals selected by stratified random sampling from 6.4 million older adults (≥ 60 years) who were followed from 2002 to 2019 [27, 28]. As a single public insurer, the NHIS covers the medical services of the entire population in South Korea. This database contains extensive medical service utilization data collected during the process of reimbursement, including patient demographic information, disease diagnoses based on the International Codes of Disease 10th Revision (ICD-10), medical services received, prescription drug records (e.g., drug codes, days of supply, and daily dosage), healthcare provider information, and results of biennial national health examinations. All the dates of medical services are also provided. The Institutional Review Board of Chung Ang University (IRB number: 1041078–201708202111-HR-322-01SB-162–01) granted an exemption from ethical review and approval for the utilization of secondary data in this study.

### Study design

**Fig. 1:**
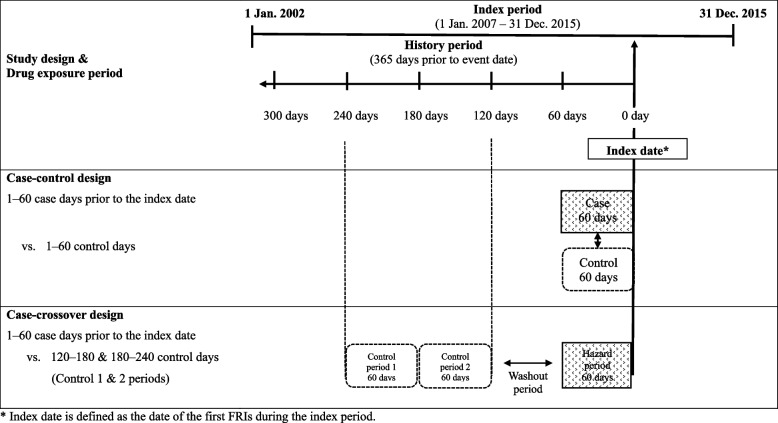
Study design

The association between medication use and the risk of FRIs was investigated using case–control and case-crossover designs to strengthen the robustness of the study results. The cases were patients with the first FRI identified by ICD-10 codes as a primary or secondary diagnosis in the claims database during the index period from January 1, 2007, to December 31, 2015. The date of the first inpatient or outpatient claims with a primary or secondary diagnosis of an FRI was assigned as the index date (Fig. 1). The history of FRIs in case patients were investigated during the history period from January 1, 2002, to December 31, 2006, to select FRI-naïve cases. Patients who did not have an FRI during the entire study period were defined as control patients. For the control patients, the index date was randomly assigned between January 1, 2007, and either the end of the study period or the date of death.

A traditional case–control design was applied to determine medications influencing the risk of FRIs; however, residual and unmeasured confounding factors after matching may still have been present. The present study conducted an additional case-crossover analysis in cases selected in the case–control analysis to compare medication exposures during hazard and control periods, which were remote times from the FRI event, within the same person. Thereby, the case-crossover design provides cases with self-controls and has the advantages of controlling for confounding by indications and unobserved between-subject confounders [24, 29, 30]. The hazard period was defined as 1–60 days prior to the index date. The two control periods were 121–180 days and 181–240 days prior to the index date. The medications prescribed on the index date were not considered to avoid potential reversal causality. A 60-day washout period was applied between the case and control periods to ensure the impact of the medication on independent control periods and to avoid carry-over effects [31]. We have chosen a 60-day drug exposure and washout period based on the health utilization pattern of older adults in Korea visiting their physicians every two months. A sensitivity analysis of the addition of washout periods between the two control periods (120–180 and 240–300 days prior to the index date) was conducted to examine the results with different timing of the control window.

### Sample selection

**Fig. 2:**
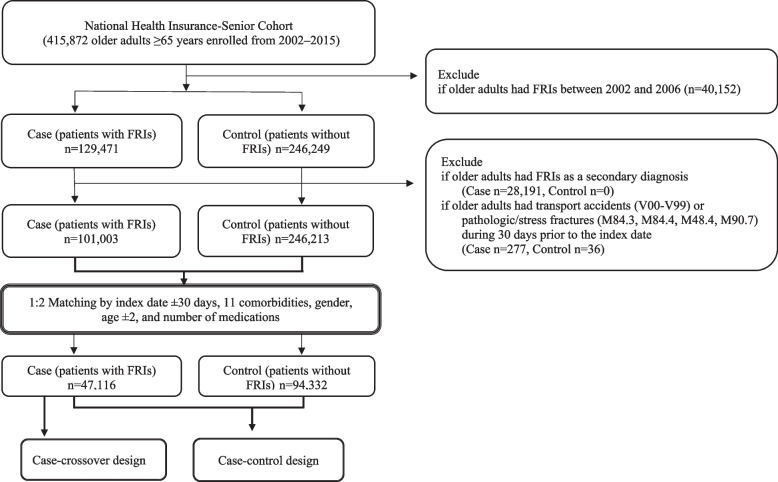
Patient selection scheme

This study identified older adults ≥ 65 years at the index date in the NHIS-senior cohort (Fig. 2). Subjects were excluded if they (a) had a history of an FRI between 2002 and 2006 or (b) had FRIs including a transport accident (ICD-10 V00-V99), pathologic fracture (M84.4, M90.7), or stress fracture (M48.4, M84.3) during the 30 days prior to the incidence of a FRI.

The eligible case patients (*n* = 101,003) were matched to control patients (*n* = 246,213) in a 1:2 ratio using the index date (± 30 days), age (± 2 years), sex, 11 comorbidities, and the number of medications used (0–2, 3–7, 8–12, 13 or more). This study mitigated the effects of bias and potential confounding with an exact matching approach [32, 33]. A total of 47,116 case patients were matched with 94,332 control patients.

Patient comorbidities that increase the risk of FRIs were selected based on previous literature and were identified using the ICD-10 codes during the 12-month history period prior to the index date [18–20]. The 11 comorbidities included cardiac arrhythmia (I44–I49), congestive heart failure (I50), hypertension (I10–I15), vestibular dysfunction and vertiginous syndrome (H81–H82), polyneuropathies and other disorders of the peripheral nervous system (G60–G64), auditory impairment (H90–H95, excluding H92), visual impairment (H25–H28, Q120, H40, H42, H53–H54), anemia (D50–64), systemic cancer (C00–C26, C30–C34, C37–41, C43, C45–C58, C60–C85, C88, C90–C97), arthritis (M05–M06, M15–M19), and transient ischemic attack and stroke (I60–I69, G45, G46, H34). The number of medications was counted by any prescriptions for the Anatomical Therapeutic Chemical (ATC) codes during the 60 days before the index date.

### Definition of an FRI

An FRI was defined based on the previous definition of serious FRIs using ICD-10 codes and ICD-10 codes mapped from ICD-9 codes [34–36]. The event was considered an FRI if any inpatient or outpatient claims with the diagnostic codes for accidental FRIs (W00–W19) were the primary or secondary diagnosis or if the injury-related codes for the primary diagnosis were (a) fall-related fractures (skull and facial bones (S02 excluding S02.5, S02.9); neck (S12); ribs, sternum and thoracic spine (S22 excluding S22.5); lumbar spine and pelvis (S32); shoulder and upper arm (S42); forearm (S52); wrist and hand (S62); femur (S72); lower leg (S82); calcaneus (S92.0); and multiple body regions (T02)), (b) intracranial injury (S06), or (c) joint dislocations ((jaw (S03.0); shoulder (S43.0); elbow (S53.0; S53.1); wrist (S63.0); knee (S83.0, S83.1); and multiple body regions (T03)).

The diagnostic codes associated with FRIs correlated well with a Korean survey study of different types of injuries associated with falls [37]. Unlike previous studies using the Medicare claims database, we did not limit the definition to emergency department visits or hospitalizations because approximately 56% of patients with FRIs visited outpatient clinics in Korea [37].

### Classification of medications

All the study medications were grouped into therapeutic classes based on the ATC classifications developed by the World Health Organization (WHO) [38]. The ATC classes that included medications used by more than 2,000 older adults in the NHIS senior cohort were selected in this study and adjusted into higher (i.e. antihypertensive) or lower levels (i.e. beta-blocking agents) of medication classes based on classifications used in previous studies (Table 2) [12–14, 17]. The asthma/chronic obstructive pulmonary disease (COPD) agents were added in this study based on the possible association of steroid inhaler usage with fracture and the use of anticholinergic medications for their indications, which can also be associated with FRIs [39, 40]. In addition, antispasmodics were also added because they can potentially increase the risk of FRIs due to their highly anticholinergic properties [39]. The final 32 subclasses of medications are listed in Table 2, and they were categorized into cardiovascular, nervous system, and other medication classes. In present study, medication subclasses will be simply referred as medications throughout the text.

### Data analyses

The differences in sociodemographic characteristics, number of concomitant medications, comorbidities, and Charlson comorbidity index (CCI) between case and control patients were assessed using t-tests, Chi-squared tests, and standardized mean differences (SMDs). The SMD is better diagnostics for large datasets to examine the balance between two groups because it is less affected by the sample size [41]. An SMD less than 0.1 is considered as well-balanced. In this case–control study, the risk of FRIs associated with each medications was examined using conditional logistic regression with and without adjustment for the other 31 medications [33, 42]. The final model only included medications with a p-value less than 0.05. In the case-crossover design, the risk of FRIs was investigated for medications that were associated with an increased risk of FRIs in the case–control design. Conditional logistic regression was conducted with and without adjustment for other medications that increased the risk of FRIs. In addition, the subgroup analysis with a case-crossover design according to the CCI (0–1, 2–4, 5 or more) was conducted to examine the risk of FRIs stratified by the severity of the patients’ conditions. All statistical analyses were conducted using R version 3.4.4 (R Foundation for Statistical Computing, Vienna, Austria) and SAS version 9.3 (SAS Institute Inc., Cary, NC, USA).

## Results

A total of 47,116 case patients were matched with 94,232 control patients. Differences in demographic characteristics, comorbidities, and CCI scores between the two groups were balanced with an SMD less than 0.1 (Table 1). The case and control patients were an average age of 71.4 years, and 56.7% were female. Table 2 presents the categorization of medication class by ATC codes. The frequencies of prescription of the 32 medications to cases and controls and the univariate analysis are presented in Table 3. The frequently used medications are in the order of nonsteroidal anti-inflammatories (NSAIDs)/antirheumatic agents, calcium channel blockers, and H2 receptor antagonists. A total of 24 medications significantly increased the risk of FRIs in the univariate analysis, and they were included in the multivariable conditional logistic regression model of the case–control design. Table 4 shows the discordant pairs of medications that patients were exposed in either the hazard or control period alone with crude odds ratios (ORs).

**Table 1:** Baseline characteristics of the study population

Characteristics	Cases (patients with FRIs), *n* = 47,116		Controls (patients without FRIs), *n* = 94,232		*p*-value	*SMD*
	**No**	**(%)**	**No**	**(%)**		
Sex						
Male	20,417	43.3%	40,834	43.3%	1	0.02
Female	26,699	56.7%	53,398	56.7%		
Age, mean (± SD)	71.4 (± 4.8)		71.3 (± 4.8)		0.003	0.02
65–69	19,784	42.0%	40,351	42.8%	0.03	0.02
70–74	15,335	32.5%	30,464	32.3%		
75–79	8,767	18.6%	17,136	18.2%		
80–84	2,928	6.2%	5,727	6.1%		
85 or more	302	0.6%	554	0.6%		
No. of medications, mean (± SD)^a^	6.7 (± 5.6)		6.5 (± 5.5)		< 0.001	0.02
0–2	12,202	25.9%	24,404	25.9%	1	0.02
3–7	17,088	36.3%	34,176	36.3%		
8–12	11,502	24.4%	23,004	24.4%		
13 or more	6,324	13.4%	12,648	13.4%		
Comorbidities^b^						
Cardiac arrhythmia	200	0.4%	400	0.4%	1	0
Congestive heart failure	135	0.3%	270	0.3%	1	0
Hypertension	24,994	53.0%	49,988	53.0%	1	0
Vestibular dysfunction and vertiginous syndrome	1271	2.7%	2542	2.7%	1	0
Polyneuropathies and other disorders of the peripheral nervous system	313	0.7%	626	0.7%	1	0
Auditory impairment	443	0.9%	886	0.9%	1	0
Visual impairment	8,695	18.5%	17,390	18.5%	1	0
Anemia	790	1.7%	1580	1.7%	1	0
Cancer	517	1.1%	1034	1.1%	1	0
Arthritis including rheumatoid arthritis	15,515	32.9%	31,030	32.9%	1	0
Transient ischemic attack and stroke	3,298	7.0%	6,596	7.0%	1	0
Charlson Comorbidity Index score, mean (± SD)	1.3 (± 1.5)		1.3 (± 1.4)		< 0.001	0.02
0–1	30,681	65.1%	62,664	66.5%	< 0.001	0.02
2–4	14,693	31.2%	28,213	29.9%		
≥ 5	1742	3.7%	3,355	3.6%		

*FRI* Fall related injuries, *No* Number, *SD* Standard deviation, *SMD* Standardized mean difference

^a^The number of concurrent medications includes all medications prescribed within 60 days prior to the index date

^b^Comorbidities were identified using ICD-10 codes during the history period

**Table 2:** Medication subclasses commonly prescribed and potentially associated with FRIs in older adults

Medication class	Subclass of the study medications	ATC-codes	Reference
Cardiovascular	ACE inhibitors	C09A-C09B, C10BX13, C10BX04, C10BX06, C10BX07, C10BX11, C10BX12, C10BX14	[11, 12, 15, 29, 35, 43]
	Angiotensin II antagonists	C09C-C09D, C10BX10	[12, 15, 29, 35, 43]
	Beta-blocking agents	C07, C09BX02	[11, 12, 15, 29, 35, 43]
	Calcium channel blockers	C08, C07FB, C09BB, C09DB, C09BX01, C09BX3, C09DX01, C09DX03, C10BX03, C10BX07, C10BX09, C10BX11, C10BX14	[12, 15, 35, 43]
	Cardiac glycosides	C01A	[11, 29, 44]
	Vasodilators	C02D, C04, C07E	[11, 12]
	Diuretics	C03, C07B- C07D, C08G, C09BA, C09DA, C09BX01, C09BX03, C09DX01, C09DX03, C10BX13	[11, 12, 15, 17, 29, 35, 45]
	Antithrombotic agents	B01, C07FX02-C07FX04, C10BX01, C10BX02, C10BX04- C10BX06, C10BX08, C10BX12	[14, 46]
	Lipid-modifying agents	C10, A10BH51	[12, 44, 46]
Nervous system	Antipsychotics	N05A	[10, 11, 13, 17, 45, 47]
	Antidepressants	N06A	[10, 11, 13, 17, 47]
	Anxiolytics	N05B	[13, 45, 47]
	Hypnotics and sedatives	N05C	[13, 17, 44, 45, 47]
	Analgesics, opioids	N02A	[10, 11, 14, 17]
	Analgesics, non-opioid	N02B, N02AJ01, N02AJ02, N02AJ03, N02AJ06, N02AJ07, N02AJ09, N02AJ13, N02AJ15, N02AJ17, N02AJ18	[14]
	Antiepileptics	N03	[10, 11, 14, 48]
	Anti-Parkinson agents	N04	[11, 14, 45]
	Anti-dementia agents	N06D	[11, 14, 47]
Others	Urological agents	G04B	[11, 39]
	Benign prostatic hypertrophy agents	G04C	[11, 44]
	Antispasmodics	A03A-A03E, A02AG, N02AG, A06AB3	[39]
	Laxatives	A06A	[11, 14]
	Antacids	A02A	[44, 45]
	H2-receptor antagonists	A02BA	[44–46]
	Proton pump inhibitors	A02BC-A02BD, M01AE52	[11, 44, 46, 49]
	Antidiabetic agents	A10	[11, 14, 44, 45]
	NSAIDs	M01A, N02AJ08, N02AJ14, N02AJ19	[11, 14, 17, 44]
	Muscle relaxants	M03B	[39, 44, 50]
	Corticosteroids (systemic)	H02A	[11, 51]
	Asthma & COPD agents	R03	[40]
	Cough and cold preparations	R05	[52]
	Antihistamines (systemic)	R06	[11, 44, 45]

**Table 3:** Univariate odds ratios of FRIs associated with medication use in older adults: case–control design

Medication class	Subclass of the study medications	Cases, *n* = 47,116		Controls, *n* = 94,232		Crude OR	95%CI	
		**No**	**(%)**	**No**	**(%)**		**Lower**	**Upper**
Cardiovascular	ACE inhibitors	1,637	3.5%	3,447	3.7%	0.95	0.89	1.01
	Angiotensin II antagonists	10,340	21.9%	22,139	23.5%	0.88	0.85	0.91
	Beta-blocking agents	5,209	11.1%	11,871	12.6%	0.85	0.82	0.88
	Calcium channel blockers	15,115	32.1%	31,134	33.0%	0.93	0.90	0.96
	Cardiac glycosides	172	0.4%	406	0.4%	0.84	0.70	1.01
	Vasodilators	3,008	6.4%	5,888	6.2%	1.03	0.98	1.08
	Diuretics	8,821	18.7%	19,287	20.5%	0.87	0.84	0.90
	Antithrombotic agents	9,533	20.2%	20,691	22.0%	0.87	0.84	0.90
	Lipid-modifying agents	8,011	17.0%	18,190	19.3%	0.83	0.81	0.86
Nervous system	Antipsychotics	744	1.6%	1,077	1.1%	1.39	1.27	1.53
	Antidepressants	2,970	6.3%	4,870	5.2%	1.25	1.19	1.31
	Anxiolytics	8,102	17.2%	14,814	15.7%	1.13	1.10	1.17
	Hypnotics and sedatives	1,556	3.3%	2,694	2.9%	1.17	1.09	1.24
	Opioids	6,304	13.4%	9,746	10.3%	1.41	1.36	1.47
	Non-opioid analgesics	7,661	16.3%	15,292	16.2%	1.00	0.97	1.04
	Antiepileptics	1,553	3.3%	2,333	2.5%	1.36	1.27	1.46
	Anti-Parkinson agents	658	1.4%	928	1.0%	1.43	1.30	1.59
	Anti-dementia agents	1,842	3.9%	3,291	3.5%	1.14	1.07	1.21
Others	Urological agents	1,121	2.4%	2,148	2.3%	1.05	0.97	1.13
	Benign prostatic hypertrophy agents	2,808	6.0%	5,908	6.3%	0.94	0.89	0.99
	Antispasmodics	5,626	11.9%	11,435	12.1%	0.98	0.95	1.02
	Laxatives	630	1.3%	1,112	1.2%	1.14	1.03	1.26
	Antacids	7,816	16.6%	14,966	15.9%	1.06	1.03	1.10
	H2-receptor antagonists	12,015	25.5%	23,217	24.6%	1.06	1.03	1.09
	Proton pump inhibitors	2,894	6.1%	5,726	6.1%	1.01	0.97	1.06
	Antidiabetic agents	6,918	14.7%	13,980	14.8%	0.99	0.96	1.02
	NSAIDs/antirheumatic agents	17,569	37.3%	31,648	33.6%	1.29	1.25	1.33
	Muscle relaxants	8,140	17.3%	11,859	12.6%	1.55	1.50	1.60
	Corticosteroids (systemic)	4,653	9.9%	8,539	9.1%	1.11	1.07	1.16
	Asthma/COPD agents	3,193	6.8%	6,849	7.3%	0.92	0.88	0.96
	Cough and cold preparations	9,055	19.2%	20,320	21.6%	0.82	0.80	0.85
	Antihistamines (systemic)	8,371	17.8%	18,408	19.5%	0.86	0.84	0.89

*FRI* Fall related injuries, *OR* Odds ratio

**Table 4:** Odds ratios of FRIs associated medication use in older adults: case-crossover design

Medication class	Subclass of the study medications	Exposed only in the hazard period, *n* = 94,332		Exposed only in the control period, *n* = 94,332		Crude OR	95%CI	
		**N**	**%**	**N**	**%**		**Lower**	**Upper**
Cardiovascular	Angiotensin II antagonists	2,148	2.3%	1,216	1.3%	1.96	1.79	2.15
	Beta-blocking agents	1,089	1.2%	1,171	1.2%	0.92	0.82	1.03
	Calcium channel blockers	2,231	2.4%	1,698	1.8%	1.38	1.27	1.50
	Diuretics	2,207	2.3%	2,127	2.3%	1.04	0.97	1.13
	Antithrombotic agents	2,204	2.3%	1,530	1.6%	1.54	1.41	1.67
	Lipid-modifying agents	2,370	2.5%	1,727	1.8%	1.46	1.34	1.58
Nervous system	Antipsychotics	420	0.4%	242	0.3%	1.84	1.51	2.25
	Antidepressants	2,110	2.2%	1,581	1.7%	1.37	1.26	1.49
	Anxiolytics	7,010	7.4%	6,159	6.5%	1.15	1.10	1.20
	Hypnotics and sedatives	1,283	1.4%	1,066	1.1%	1.22	1.10	1.35
	Opioids	7,642	8.1%	5,015	5.3%	1.56	1.49	1.63
	Antiepileptics	1,342	1.4%	1,016	1.1%	1.37	1.23	1.52
	Anti-Parkinson agents	259	0.3%	149	0.2%	1.88	1.45	2.43
	Anti-dementia agents	1,072	1.1%	945	1.0%	1.16	1.03	1.30
Others	Benign prostatic hypertrophy agents	1,387	1.5%	1,095	1.2%	1.31	1.18	1.45
	Antacids	8,287	8.8%	8,057	8.6%	1.03	0.99	1.07
	H2-receptor antagonists	11,569	12.3%	10,306	10.9%	1.13	1.09	1.17
	Laxatives	804	0.9%	701	0.7%	1.16	1.02	1.32
	NSAIDs/antirheumatic agents	16,069	17.1%	12,328	13.1%	1.32	1.28	1.36
	Muscle relaxants	10,442	11.1%	6,513	6.9%	1.65	1.59	1.72
	Corticosteroids (systemic)	5,812	6.2%	5,589	5.9%	1.04	1.00	1.09
	Asthma/COPD agents	3,516	3.7%	3,735	4.0%	0.94	0.89	1.00
	Cough and cold preparations	10,691	11.3%	11,923	12.7%	0.89	0.87	0.92
	Antihistamines (systemic)	9,861	10.5%	10,786	11.4%	0.91	0.88	0.94

*FRI* Fall related injuries, *OR* Odds ratio

### Association of nervous system and cardiovascular medications with the risk of FRIs

**Fig. 3:**
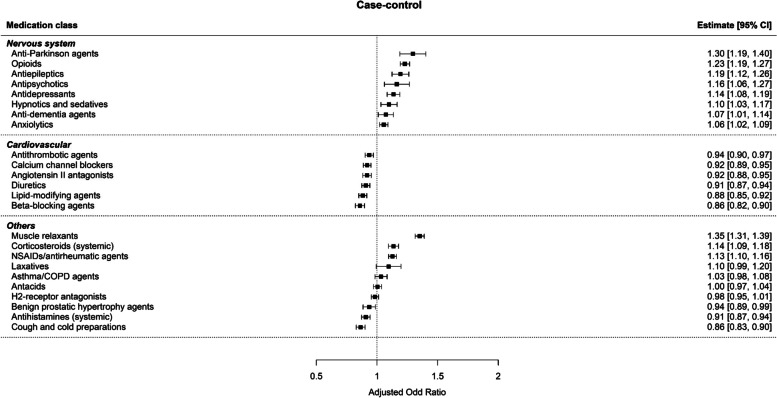
Risk of FRIs associated with medications from the case–control design. The odds ratio of each medication subclass was adjusted with the other 23 medication subclasses included in the model

**Fig. 4:**
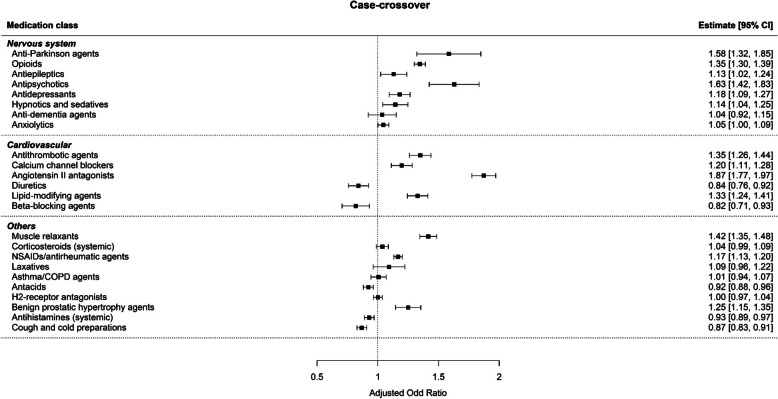
Risk of FRIs associated with medications from the case-crossover design. The odds ratio of each medication was adjusted with the other 23 medications included in the model

The increased risk of FRIs was consistent in following nervous system medications from both study designs: anti-Parkinson agents (Adjusted Odd Ratio[AOR] 1.30; 95% Confidence Interval[CI] 1.19–1.40), opioids (AOR 1.23; 95%CI 1.19–1.27), antiepileptics (AOR 1.16; 95%CI 1.12–1.26), antipsychotics (AOR 1.14; 95%CI 1.06–1.27), antidepressants (AOR 1.10; 95%CI 1.03–1.17), hypnotics and sedatives (AOR 1.01; 95%CI 1.03–1.17), and anxiolytics (AOR 1.06; 95%CI 1.02–1.09) (Figs. 3 and 4). However, anti-dementia agents were not associated with FRIs in the case-crossover design (AOR 1.04; 95%CI 0.92–1.15). The subgroup analysis by CCI also showed that anti-dementia agents was not associated with FRIs regardless of patients’ comorbid conditions (Supplementary Table 1).

While the risk of FRIs with nervous system medications was mostly consistent between the case–control and case-crossover studies, cardiovascular medications showed conflicting results. All cardiovascular medications did not increase the risk of FRIs in the case–control design. In case-crossover design, antithrombotic agents (AOR 1.35; 95%CI 1.26–1.44), calcium channel blockers (AOR 1.20; 95%CI 1.11–1.28), angiotensin II antagonists (AOR 1.87; 95%CI 1.77–1.97), and lipid-modifying agents (AOR 1.33; 95%CI 1.24–1.41) increased the risk of FRIs. In the subgroup analysis, the risk of FRIs with the use of antithrombotic agents, calcium channel blockers, angiotensin II antagonists, and lipid-modifying agents was higher in the CCI 0–1 group than in the CCI 2–4 group.

### Association of other medications with the risk of FRIs

The risks of FRI with other medications were significantly increased in both the case–control and case-crossover studies for muscle relaxants (AOR 1.35; 95% CI 1.31–1.39 and AOR 1.42; 95%CI 1.35–1.48, respectively) and NSAIDs/antirheumatic agents (AOR 1.13; 95%CI 1.10–1.16 and AOR 1.17; 95%CI 1.13–1.20, respectively). Corticosteroids were associated with an increased risk of FRIs in the case–control study but not in the case-crossover study (AOR 1.14; 95% CI 1.09–1.18 and AOR 1.04; 95%CI 0.99–1.09, respectively). Furthermore, benign prostatic hypertrophy agents were associated with an increased risk of FRIs in the case-crossover design only (AOR 1.25; 95%CI 1.15–1.35).

### Sensitivity analysis with different control periods

Most medications showed a similar risk of FRIs in the sensitivity analysis of different control periods (120–180 and 240–300 days prior to the index date) compared with the main analysis (Supplementary Table 2). The risk of anti-Parkinson agents, opioids, antipsychotics, antidepressants, hypnotics and sedatives, anti-dementia agents on FRIs was consistent. Antiepileptics, anxiolytics, and hypnotics and sedatives did not increase the risk of FRIs in the sensitivity analysis. All cardiovascular medications maintained same association with FRIs as the main analysis. Among other classes of medications, the sensitivity analysis reported the corticosteroids and laxatives use increased the risk of FRIs.

## Discussion

This population-based study confirmed that anti-Parkinson agents, opioids, antiepileptics, antipsychotics, antidepressants, hypnotics and sedatives, anxiolytics, muscle relaxants, and NSAIDs/antirheumatic agents increased the risk of FRIs in both the case–control and case-crossover study designs. Some of the cardiovascular medications (antithrombotic agents, calcium channel blockers, angiotensin II antagonists, lipid-modifying agents) and benign prostatic hypertrophy agents were associated with an increased risk of FRIs only in the case-crossover design. This suggests a potential transient effect of medications to increase the risk of FRIs that was captured in the case-crossover study. On the other hand, corticosteroids were only found to increase the risk of FRIs in the case–control design, which indicates the cumulative effects of corticosteroids on FRIs.

Nervous system medications are known to be associated with FRIs, and our results was consistent with previous meta-analyses and guidelines [10, 11, 13, 14]. Nervous system medications have adverse events such as dizziness, sedation, and decreased cognitive function, which can increase the risk of FRIs in older adults. For example, antidepressants have adverse events of reduced cognitive function, orthostatic hypotension, sleep disturbances, sedation, and anticholinergic activities that can lead to FRIs [35, 53, 54]. Benzodiazepines are also associated with confusion, dizziness, and sedation, which can increase the risk of FRIs [35, 53, 54]. However, the sensitivity analysis in case-crossover design with different control periods reported inconsistent results with antiepileptics, anxiolytics, and hypnotics and sedatives. These medications showed no association with FRIs after adjusting for other concurrent medications. Further studies should be conducted considering concurrent medications is needed to have deeper understanding of the risk of FRIs in these medications.

Anti-Parkinson agents were associated with a high risk of FRIs in both the case–control and case-crossover studies. A previous meta-analysis found controversial results that the fall risk was not increased by anti-Parkinson agents (pooled OR: 1.54; 95% CI 0.95–2.43) [14]. Since Parkinson’s disease itself is a risk factor for FRIs and patients with Parkinson’s disease must take anti-Parkinson agents, it is hard to distinguish the effects of medications from their indications in traditional case–control studies [20]. The results of this case-crossover study confirmed the increased risk of FRIs with anti-Parkison’s agents because this design controls for confounding by indications by self-comparison. Another case-crossover study conducted in Japan also showed an increased risk of inpatient falls with anti-Parkinson agents [45]. Anti-dementia medications are also similar to anti-Parkinson agents in that all patients with dementia will eventually take these medications for the rest of their lives, and dementia is also a risk factor for FRIs [18, 20]. However, anti-dementia agents were not associated with FRIs in this case-crossover design. The risk of FRIs with anti-dementia agents appeared to be not transient.

Our study found that cardiovascular medications were associated with a reduced risk of FRIs in case–control design, but some of them (antithrombotic agents, calcium channel blockers, angiotensin II antagonists, lipid-modifying agents) rather increased the risk in the case-crossover design. Previous studies have found conflicting effects of antihypertensive medications on the risk of FRIs, showing no association with FRIs and an increased risk of FRIs [12, 15, 17, 29, 35]. These conflicting results may be due to different durations of drug utilization. All antihypertensive drug categories were associated with an increased risk of FRIs within the first 15 days of drug use in both self-controlled case series and case-crossover studies [22, 43]. Antihypertensive medications can increase the risk of FRIs at the initiation by the first-dose effect causing orthostatic hypotension [23]. Therefore, our case-crossover design reflected an increasing risk with the initiation of calcium channel blockers and angiotensin II antagonists.

In the case-crossover study, the increased risk of FRIs with antithrombotic agents and lipid modifying agents was also unexpected based on the findings of previous studies. There are no known mechanisms for FRIs with antithrombotic agents and lipid-modifying agents. A meta-analysis and previous observational studies also found that lipid-modifying agents or statins were associated with a reduced risk of falls or fractures [12, 55, 56]. The increased risk of FRIs with benign prostatic hypertrophy agents indicates that alpha blockers or 5-reducate inhibitors may be associated with a higher risk at the time of initiation. Steroids, on the other hand, were only associated with FRIs in the case–control study, indicating a cumulative effect of steroids on fracture risk [57]. The corticosteroids and laxatives were not associated with FRIs after adjusting with other concurrent medications and they showed association in the sensitivity analysis, which suggest the potential impact of concurrent medications on FRIs.

To the best of our knowledge, this is the first study to investigate risk of FRIs associated with medications commonly prescribed for older adults in Korea. This study was conducted using a large nationwide insurance claims database that is representative of older adults with long-term data from 2002 to 2015. Therefore, recall and selection bias were limited, and the results are generalizable to older Korean adults. Second, we minimized confounding errors through the study design. A strict matching scheme was applied in this study to remove other risk factors associated with FRIs, thereby focusing on the medications. Also, a case-crossover design was applied for adjustments of residual confounders from case–control design. The case-crossover design, however, does not control for time-variant confounders; thus, we attempted to have shorter period between controls and periods, assuming consistent health during that period. Furthermore, adjustment by other medications facilitated to control for time-variant confounders by accounting for concurrent medications prescribed during hazard and control periods.

Despite this study’s strengths, there are some limitations that warrant further consideration. First, the claims database has an inherent limitation of not including detailed clinical or demographic information, which are not collected for the reimbursement process. For example, gait abnormalities, balance impairments, impaired activities of daily living, cognitive impairments, use of assistive devices, living status (living alone), and environmental hazards, are not captured in the claims database but also important risk factors that might affect the results [18–20]. This study controlled for those confounders by employing the case-crossover design with self-comparison. The date of actual intake was implausible in the claims database. Thus, the misclassification bias of exposure could have impacted the results. However, such misclassification bias can be considered minimal because most of patients in Korea visit the pharmacy on the same date as the medication is prescribed. Second, although the previously validated definition of an FRI was adapted, this is the first study to identify this event using a Korean claims database and the misclassifications can occur. The current definition of an FRI may not capture less severe cases of falls without injuries in older adults.

Third, the case-crossover design investigated the risk of FRIs in all medications regardless of their use in the short or long term. A case-crossover design is not necessarily appropriate for long-term medications due to persistent user bias. A simulation study of the case-crossover method suggested upward bias occurred with persistent users; however, the estimated effect did not vary substantially to the magnitude of the true effect [26, 30]. We expect that the current study design may have had upward bias, but the findings are still plausible to explain the true effects of medications on FRIs. corticosteroids and laxatives use increased the risk of FRIs.

## Conclusions

This population-based study investigated the robust association of medications including anti-Parkinson agents, opioids, antiepileptics, antipsychotics, antidepressants, hypnotics and sedatives, anxiolytics, muscle relaxants, and NSAIDs/antirheumatic agents with the risk of FRIs in older adults using case–control and case-crossover designs. Antithrombotic agents, calcium channel blockers, angiotensin II antagonists, lipid-modifying agents, and benign prostatic hypertrophy agents were only associated with an increased risk of FRIs in the case-crossover design and potentially have a transient effect on FRIs at the time of their initiation. Corticosteroids, however, increased the risk of FRIs only in case–control design, indicating the cumulative effects of corticosteroids on FRIs.

## Supplementary Information

**Additional file 1: Supplementary Table 1.** Subgroup analysis of the case-crossover design by CCI (0–1, 2–4, 5 or more). **Supplementary Table 2.** Sensitivity analysis of the case-crossover design with an additional washout period (control periods: 120–180 and 240–300 days prior to the index date).

## Data Availability

The NHIS senior cohort is available upon permission from the NHIS (https://nhiss.nhis.or.kr) after the committee review.
