# Supplementary material for: Medications influencing the risk of fall-related injuries in older adults: case–control and case-crossover design studies
Source: BMC Geriatr. 2023 Jul 22;23:452. doi: 10.1186/s12877-023-04138-z (PMC10363319; doi:10.1186/s12877-023-04138-z)
Supplement: Supplementary file 1 — Additional file 1: Supplementary Table 1. Subgroup analysis of the case-crossover design by CCI (0–1, 2–4, 5 or more). Supplementary Table 2. Sensitivity analysis of the case-crossover design with an additional washout period (control periods: 120–180 and 240–300 days prior to the index date). [file 12877_2023_4138_MOESM1_ESM.docx]

# Supplementary Table 1. Subgroup analysis of case-crossover design by Charlson comorbidity index (0–1, 2–4, 5 or more)

| **Medication class** | **Subclass of the study medication** | **0**–**1** | | | **2**–**4** | | | **5 or more** | | |
| --- | --- | --- | --- | --- | --- | --- | --- | --- | --- | --- |
|  |  | **AOR** | **95%CI**  **Lower** | **95%CI Upper** | **AOR** | **95%CI**  **Lower** | **95%CI Upper** | **AOR** | **95%CI**  **Lower** | **95%CI Upper** |
| Nervous system | Anti-Parkinson agents | 1.29 | 0.855 | 1.946 | 1.555 | 1.054 | 2.293 | 3.169 | 1.447 | 6.937 |
|  | Opioids | 1.413 | 1.327 | 1.504 | 1.256 | 1.167 | 1.352 | 1.388 | 1.136 | 1.696 |
|  | Antiepileptics | 1.103 | 0.941 | 1.293 | 1.159 | 0.989 | 1.357 | 1.306 | 0.903 | 1.89 |
|  | Antipsychotics | 1.855 | 1.353 | 2.542 | 1.242 | 0.912 | 1.691 | 2.987 | 1.667 | 5.354 |
|  | Antidepressants | 1.038 | 0.912 | 1.183 | 1.33 | 1.176 | 1.505 | 1.149 | 0.868 | 1.521 |
|  | Hypnotics and sedatives | 1.09 | 0.939 | 1.266 | 1.247 | 1.066 | 1.459 | 0.938 | 0.647 | 1.358 |
|  | Anti-dementia agents | 0.88 | 0.732 | 1.059 | 1.174 | 0.996 | 1.383 | 1.154 | 0.805 | 1.654 |
|  | Anxiolytics | 1.052 | 0.989 | 1.118 | 1.01 | 0.941 | 1.084 | 1.305 | 1.068 | 1.593 |
| Cardiovascular | Antithrombotic agents | 1.402 | 1.212 | 1.621 | 1.377 | 1.217 | 1.558 | 1.088 | 0.825 | 1.436 |
|  | Calcium channel blockers | 1.282 | 1.136 | 1.446 | 1.086 | 0.949 | 1.243 | 1.141 | 0.846 | 1.538 |
|  | Angiotensin II antagonists | 2.144 | 1.852 | 2.481 | 1.814 | 1.568 | 2.099 | 1.114 | 0.803 | 1.545 |
|  | Diuretics | 0.847 | 0.749 | 0.958 | 0.805 | 0.71 | 0.912 | 0.982 | 0.74 | 1.302 |
|  | Lipid-modifying agents | 1.447 | 1.271 | 1.677 | 1.21 | 1.072 | 1.365 | 1.529 | 1.15 | 2.033 |
|  | Beta-blocking agents | 0.783 | 0.665 | 0.923 | 0.851 | 0.716 | 1.01 | 0.838 | 0.589 | 1.193 |
| Others | Muscle relaxants | 1.453 | 1.371 | 1.54 | 1.341 | 1.249 | 1.44 | 1.526 | 1.251 | 1.863 |
|  | Corticosteroids (systemic) | 1.012 | 0.946 | 1.082 | 1.079 | 0.998 | 1.167 | 0.895 | 0.716 | 1.119 |
|  | NSAIDs/antirheumatic agents | 1.203 | 1.15 | 1.259 | 1.112 | 1.048 | 1.179 | 1.077 | 0.908 | 1.279 |
|  | Laxatives | 1.056 | 0.857 | 1.302 | 1.153 | 0.953 | 1.395 | 0.976 | 0.683 | 1.395 |
|  | Asthma/COPD agents | 0.96 | 0.874 | 1.055 | 1.03 | 0.941 | 1.127 | 1.108 | 0.871 | 1.408 |
|  | Antacids | 0.927 | 0.878 | 0.979 | 0.909 | 0.852 | 0.969 | 1.006 | 0.839 | 1.205 |
|  | H2-receptor antagonists | 1.008 | 0.961 | 1.057 | 0.996 | 0.94 | 1.056 | 0.905 | 0.766 | 1.069 |
|  | Benign prostatic hypertrophy agents | 1.2 | 1.036 | 1.389 | 1.344 | 1.148 | 1.575 | 1.023 | 0.695 | 1.506 |
|  | Antihistamines (systemic) | 0.954 | 0.904 | 1.007 | 0.897 | 0.841 | 0.957 | 0.94 | 0.782 | 1.13 |
|  | Cough and cold preparations | 0.847 | 0.803 | 0.893 | 0.885 | 0.831 | 0.943 | 0.974 | 0.815 | 1.163 |

AOR: Adjusted Odd Ratio

# Supplementary Table 2. Sensitivity analysis of case-crossover design with additional wash-out period (control periods: 120–180 and 240–300 days prior to the index date­)

| **Medication class** | **Subclass of the study medication** | **Exposed only in the hazard period,**  **n=94.332** | | **Exposed only in the control period,**  **n=94,332** | | **Crude odd ratios** | **95%CI** | | **Adjusted odd ratios** | **95%CI** | |
| --- | --- | --- | --- | --- | --- | --- | --- | --- | --- | --- | --- |
|  |  | **N** | **%** | **N** | **%** |  | **Lower** | **Upper** |  | **Lower** | **Upper** |
| Nervous system | Anti-Parkinson agents | 279 | 0.3% | 158 | 0.2% | 1.88 | 1.45 | 2.43 | 1.57 | 1.23 | 2.00 |
|  | Opioids | 7,722 | 8.2% | 5,070 | 5.4% | 1.56 | 1.49 | 1.63 | 1.33 | 1.27 | 1.39 |
|  | Antiepileptics | 1,405 | 1.5% | 1,078 | 1.1% | 1.37 | 1.23 | 1.52 | 1.09 | 0.99 | 1.20 |
|  | Antipsychotics | 453 | 0.5% | 257 | 0.3% | 1.84 | 1.51 | 2.25 | 1.61 | 1.33 | 1.96 |
|  | Antidepressants | 2,207 | 2.3% | 1,628 | 1.7% | 1.37 | 1.26 | 1.49 | 1.20 | 1.11 | 1.30 |
|  | Hypnotics and sedatives | 1,328 | 1.4% | 1,151 | 1.2% | 1.22 | 1.10 | 1.35 | 1.08 | 0.98 | 1.19 |
|  | Anti-dementia agents | 1,188 | 1.3% | 1,025 | 1.1% | 1.16 | 1.03 | 1.30 | 1.06 | 0.95 | 1.18 |
|  | Anxiolytics | 7,087 | 7.5% | 6,344 | 6.7% | 1.15 | 1.10 | 1.20 | 1.02 | 0.98 | 1.07 |
| Cardiovascular | Antithrombotic agents | 2,463 | 2.6% | 1,644 | 1.7% | 1.54 | 1.41 | 1.67 | 1.36 | 1.26 | 1.48 |
|  | Calcium channel blockers | 2,473 | 2.6% | 1,845 | 2.0% | 1.38 | 1.27 | 1.50 | 1.19 | 1.10 | 1.29 |
|  | Angiotensin II antagonists | 2,416 | 2.6% | 1,297 | 1.4% | 1.96 | 1.79 | 2.15 | 1.87 | 1.71 | 2.04 |
|  | Diuretics | 2,401 | 2.5% | 2,313 | 2.5% | 1.04 | 0.97 | 1.13 | 0.84 | 0.77 | 0.90 |
|  | Lipid-modifying agents | 2,618 | 2.8% | 1,833 | 1.9% | 1.46 | 1.34 | 1.58 | 1.35 | 1.25 | 1.46 |
|  | Beta-blocking agents | 1,183 | 1.3% | 1,323 | 1.4% | 0.92 | 0.82 | 1.03 | 0.80 | 0.72 | 0.89 |
| Others | Muscle relaxants | 10,615 | 11.3% | 6,650 | 7.1% | 1.65 | 1.59 | 1.72 | 1.39 | 1.33 | 1.45 |
|  | Corticosteroids (systemic) | 5,855 | 6.2% | 5,585 | 5.9% | 1.04 | 1.00 | 1.09 | 1.05 | 1.00 | 1.10 |
|  | NSAIDs/antirheumatic agents | 16,325 | 17.3% | 12,550 | 13.3% | 1.32 | 1.28 | 1.36 | 1.16 | 1.13 | 1.20 |
|  | Laxatives | 828 | 0.9% | 677 | 0.7% | 1.16 | 1.02 | 1.32 | 1.17 | 1.03 | 1.33 |
|  | Asthma/COPD agents | 3,549 | 3.8% | 3,819 | 4.1% | 0.94 | 0.89 | 1.00 | 1.00 | 0.94 | 1.07 |
|  | Antacids | 8,393 | 8.9% | 8,264 | 8.8% | 1.03 | 0.99 | 1.07 | 0.91 | 0.88 | 0.95 |
|  | H2-receptor antagonists | 11,792 | 12.5% | 10,460 | 11.1% | 1.13 | 1.09 | 1.17 | 1.01 | 0.98 | 1.05 |
|  | Benign prostatic hypertrophy agents | 1,489 | 1.6% | 1,172 | 1.2% | 1.31 | 1.18 | 1.45 | 1.24 | 1.13 | 1.36 |
|  | Antihistamines (systemic) | 9,879 | 10.5% | 10,848 | 11.5% | 0.91 | 0.88 | 0.94 | 0.93 | 0.90 | 0.97 |
|  | Cough and cold preparations | 10,578 | 11.2% | 11,975 | 12.7% | 0.89 | 0.87 | 0.92 | 0.86 | 0.83 | 0.89 |
